# On the stability of morphology and performance of neural interfacing electrodes fabricated via CO_2_-snow-assisted hierarchical surface restructuring

**DOI:** 10.1371/journal.pone.0349598

**Published:** 2026-07-09

**Authors:** Alexander Blagojevic, Wesley Seche, Pouya Tavousi, Sina Shahbazmohamadi, Shahram Amini

**Affiliations:** 1 Department of Biomedical Engineering, University of Connecticut, Storrs, Connecticut, United States of America; 2 Pulse Technologies Inc. (An Integer Holdings Company), Research & Development, Quakertown, Pennsylvania, United States of America; 3 Tescan Orsay Holding, Libušina tř., Brno-Kohoutovice, Czechia; Universidad Tecnica de Ambato, ECUADOR

## Abstract

Long-term implantable neural interfacing devices play a critical role in treating various neurological disorders, with their functionality largely dependent on the performance of electrodes and microelectrode arrays. Femtosecond laser Hierarchical Surface Restructuring (HSR™) is an advanced surface treatment technology that significantly enhances a platinum-10% iridium (Pt-10Ir) electrode’s electrochemical performance, improving energy efficiency, specificity, and signal-to-noise ratio. Additionally, HSR™ facilitates electrode miniaturization, allowing them to be manufactured smaller, for a less invasive profile. Electrode surfaces produced via HSR™ technology contain multiscale structures, including nanoscale features that, while contributing to superior performance, are sometimes weakly bonded and may detach due to mechanical agitation during testing or implantation. This detachment could lead to a high initial performance, that may gradually decline during prolonged use. Preemptively removing these nanostructures stabilizes the electrode surface, enhancing the stability of its morphology and potentially electrochemical performance. This study introduces a novel, in-operando, CO₂-snow-assisted HSR™ process and benchmarks it against other prevalent surface cleaning methods post-fabrication, such as ultrasonic cleaning, on improving electrode stability and performance. Both qualitative and quantitative analyses indicate that all cleaning methods enhance electrode stability. However, ultrasonic cleaning was found to be more destructive compared to CO₂-snow-assisted HSR™ processing, resulting in reduced electrochemical performance. In contrast, in-operando CO₂-snow-assisted processing provided similar or superior improvements in surface stability, while preserving higher electrochemical performance *in vitro* and enabling a faster processing time. This study is the gateway to further assess the stability *in vivo*, which is the intended next step of the research.

## Introduction

**Fig 1:**
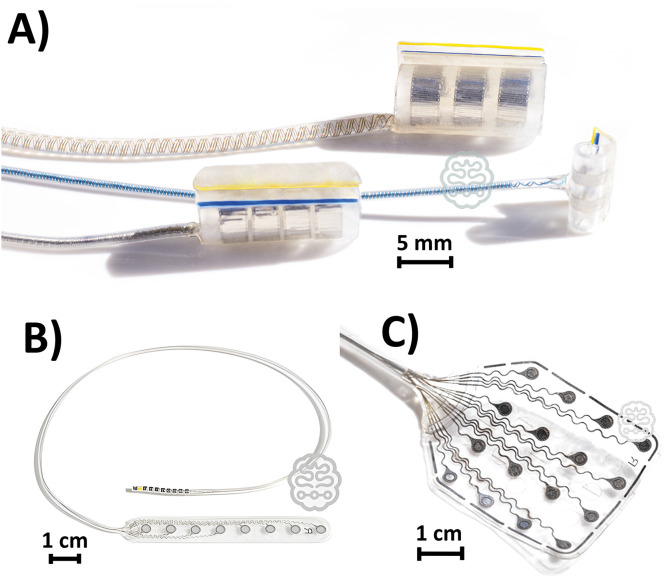
Examples of various geometries and arrangements of commercially available electrode arrays used for recording and stimulation of neural tissue. CorTec® °AirRay® **(A)** cuff, **(B)** cortical strip, and **(C)** cortical grid electrode arrays with Pt-10Ir electrode contacts used for stimulation and recording of nerve tissue (images used with permission and courtesy of CorTec®).

Implantable neural interfacing devices are essential for diagnosing and treating a wide range of cardiac, neurological, retinal, and auditory disorders. These devices interface directly with the nervous systems, either delivering targeted electrical stimulation to modulate neural activity, or recording neuronal signals to assess and manage various neurological disorders. Their effectiveness depends on the precision, stability, and long-term reliability of the electrodes and microelectrode arrays that form the core of these devices [1]. Examples of these implantable devices include neurostimulation systems such as spinal cord stimulators, sacral nerve stimulators, vagal nerve stimulators, deep brain stimulators, and responsive neurostimulators [2–5]. Additionally, cardiac rhythm management devices, Cochlear implants and retinal and bionic vision prosthesis play a crucial role in restoring lost physiological functions [6–8]. Some representative examples of these devices are illustrated in **Fig 1**.

Electrodes and microelectrode arrays serve as the critical interface between the device and target neural tissue. Their design incorporates various materials, interface geometries, and electrochemical properties tailored to the specific requirements of their applications. In both stimulation and recording contexts, high-density electrode arrays are particularly advantageous, as they enable more precise targeting of discrete neuronal populations. This enhanced selectivity improves signal fidelity, spatial resolution, and the overall efficacy of neurostimulation and sensing/recording systems, ultimately leading to more robust control and monitoring of biological responses [1,9–11].

The development of high-density microelectrode arrays requires smaller electrode contacts. However, further miniaturization has been constrained by the limitations of existing manufacturing techniques [12]. Regardless of the application, high-performance electrodes are typically characterized by low impedance, high charge storage capacity (CSC), and high specific capacitance (SC) [1]. While reducing electrode size enhances spatial selectivity of neural interfacing, it also decreases the geometric surface area (GSA) of the electrode contacts, leading to an unavoidable tradeoff between spatial selectivity and properties that enable favorable electrode performance. This can degrade electrode functionality undermining the benefits of improved selectivity.

To address this tradeoff and preserve both high selectivity and electrochemical performance while maintaining a low GSA, various surface modification techniques have been developed. These include thin-films and coatings [13–15] as well as physical and electrochemical surface treatments designed to enhance surface roughness [16] thereby increasing the electrochemically active surface area (ESA) independently of the GSA. A higher ESA allows for improved charge transfer efficiency and electrochemical performance per unit area. By optimizing the ESA/GSA ratio, electrodes can be densely packed within a device to improve spatial selectivity, while maintaining high signal fidelity, efficient power consumption, and robust performance [1].

Femtosecond laser Hierarchical Surface Restructuring (HSR™) is one such surface modification technologies. In previous studies, HSR™ has demonstrated significant improvements in the performance of platinum-10% (by weight) iridium alloy (Pt-10Ir) electrodes, increasing CSC by two orders of magnitude and enhancing specific capacitance by more than 700-fold. This approach enables significant electrochemical enhancements while maintaining electrode miniaturization, making it highly suitable for advanced neural interfacing applications [17–19].

The HSR™ technique employs a femtosecond laser beam to restructure the surface into a pattern of periodic, pillar-shaped, hierarchical structures, characterized by multiple levels of surface features spanning across distinct length scales. This unique topography significantly enhances the ESA independently of GSA that results in a high ESA/GSA ratio, critical for improving electrode performance while preserving miniaturization.

It is well-established that during the fabrication of an HSR™ electrode, ablated material from the electrode surface may redeposit onto the surface, forming micron- to nanometer-scale particulates [17]. Among these, the most commonly observed are nanoscale, snowflake-like particles that densely coat the restructured surface.

These particles exhibit a high surface area, which enhances the ESA and overall electrode performance [17,20,21]. However, they appear to be weakly bonded to the substrate and are prone to detachment under mechanical or electrochemical agitation, such as during cyclic voltammetry (CV) testing. This detachment can lead to gradual morphological changes over time, potentially affecting electrode stability. This instability is undesirable, as it may result in an initially high electrode performance, that diminishes over time as these relatively loose nanoparticles detach during testing or use.

To enhance the stability and consistency of electrode performance, these nanoparticles can be preemptively removed through a controlled cleaning process. This approach ensures that only the most securely bonded surface structures remain, minimizing morphological changes and performance degradation over time. By eliminating weakly attached particulates before implantation or testing, the electrode’s long-term reliability and electrochemical stability can be significantly improved.

One widely used cleaning method involves submerging the electrodes in a liquid (typically an alcohol or solvent) while applying ultrasonic frequency sound waves. The vibrations produced by this process effectively remove contaminants and dislodge loose particles from the surface, ensuring a cleaner and more stable electrode interface. In the context of cleaning methods, “loose” refers to a surface feature’s relative lack of resistance to being removed by a cleaning process. However, the potential impact of this method on underlying electrode morphology and electrochemical performance warrants further investigation [22]. While ultrasonic cleaning is effective in removing unstable surface structures, we will demonstrate in this report that it is excessively aggressive, leading to unintended surface damage. This excessive removal can degrade key morphological features of the electrode, ultimately resulting in a significant reduction in electrochemical performance. Ultrasonic cleaning also requires additional post processing steps, which will increase fabrication time.

An alternative cleaning technique is CO₂-snow-assisted processing, in which gaseous carbon dioxide is compressed to its triple point and expelled as a high-velocity jet, containing dry ice particles, as well as liquid and gaseous CO₂. This process effectively removes particulates and thin-film contaminants, including relatively loose nanoparticles and other unstable surface structures, without introducing additional residues. Unlike liquid-based cleaning methods, CO₂-snow-assisted processing is a non-abrasive approach that preserves the underlying electrode morphology while ensuring a clean and stable surface [23,24]. During HSR™ electrode fabrication, CO₂-snow-assisted processing is performed in-operando during the femtosecond laser manufacturing process, using a gas nozzle that is aligned with the laser focal point. This setup enables real-time processing, allowing particulate removal to occur simultaneously during HSR™ electrode fabrication, adding little to no additional processing time. Additionally, CO₂-snow-assisted processing can be applied immediately after restructuring to ensure thorough removal of unstable surface structures without interfering with the laser ablation process.

### Objectives

The objective of this study is to evaluate the feasibility and benefits of integrating CO₂-snow-assisted processing into the fabrication process of femtosecond laser HSR™ Pt-10Ir electrodes for neural interfacing applications. Specifically, this work examines the impact of surface cleaning on Pt-10Ir HSR™ electrodes and compares the effectiveness of ultrasonic cleaning and CO₂-snow-assisted processing in removing unstable surface structures while preserving electrode morphology and electrochemical performance. While various other electrode cleaning methods exist, many are either primarily designed for sterilization with minimal mechanical interaction—such as autoclaving, UV exposure, and chemical sterilization—or are excessively abrasive, like scrubbing, which can destroy the relatively delicate HSR™ electrode surfaces.

In this study, we assess the efficacy of each surface cleaning technique in removing unstable surface structures, by evaluating the improvement to the stability of electrode morphology. We aim to identify an optimal method(s) that enhances electrode stability, without compromising electrochemical performance. Here, stability is defined as the electrode surface’s resistance to change or alteration when subjected to mechanical or electrochemical agitation. This will be characterized through qualitative analysis of SEM micrographs and quantitative surface analysis, before and after agitation.

Additionally, we investigate the underlying mechanisms driving morphological changes induced by different processing methods, assessing their potential benefits and drawbacks. To comprehensively evaluate these effects, each cleaning technique is tested on electrodes fabricated at varying laser fluence levels, allowing us to assess how surface morphology and electrochemical performance influence cleaning effectiveness across electrodes with different morphologies and performance levels.

## Materials and methods

### Electrode Fabrication via Femtosecond Laser Hierarchical Surface Restructuring (HSR™)

The electrodes were fabricated using a FemtoChisel System (Tescan, Storrs, Connecticut, USA) with a Monaco 1035 femtosecond laser source (Coherent, Santa Clara, CA, USA), which produces 257 fs pulses at a central wavelength of 1035 nm. Beam deflection and targeting were controlled via an IntelliSCAN galvo scan head (SCANLAB, Pucheim, Germany), ensuring precise patterning of the electrode surface for hierarchical surface restructuring. The experiments were conducted in an ambient air environment. Electrodes were securely mounted on a porous ceramic vacuum chuck equipped with three-dimensional stage controls (Zaber, Vancouver, BC, Canada) to ensure precise positioning. To maintain accurate focus, the electrodes were leveled with the focal plane of the scan head using a Keyence Confocal Displacement Sensor (Keyence, Osaka, Japan). Targeting and alignment of the laser pattern were verified using a digital microscope (IDS, Massachusetts, USA).

HSR™ electrodes are categorized based on the laser fluence used during fabrication, as fluence is a key parameter that directly influences surface morphology and electrochemical performance. Fluence, defined as the energy delivered per unit area, is expressed in J/cm². For this study, nine 5 mm × 5 mm (125 µm thick) Pt-10Ir electrodes were fabricated for each of the three processing and cleaning methods tested, at three distinct laser fluences: 1.8 J/cm², 3.6 J/cm², and 9.7 J/cm². At each fluence, three identical electrodes were produced to ensure repeatability. Additionally, a control group of nine electrodes per fluence was fabricated, which did not undergo any cleaning process, allowing for direct comparison of surface stability and performance.

### Cleaning Methods

**Fig 2:**
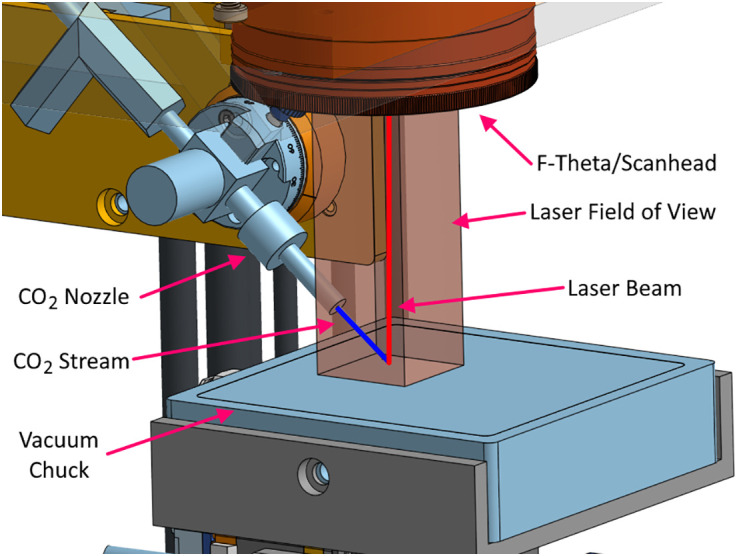
Diagram of CO_2_ processing setup, showing f-theta focusing lens (top), CO_2_ nozzle (center-left), and vacuum mounting chuck (bottom). The red line shows the laser beam path, the transparent red rectangle represents the scan field-of-view of the laser, and the blue line represents the path of the CO_2_ stream.

The CO₂ nozzle is mounted beneath the scan head, positioned to the side of the stage mount, and calibrated to direct its flow precisely at the laser-electrode interaction point. The nozzle is angled at approximately 30° relative to the stage mount (**Fig 2**), ensuring effective removal of ablated particulates in real time during the hierarchical surface restructuring process [24,25]. This setup allows for in-operando CO₂ snow-assisted processing, minimizing residual debris while preserving the integrity of the restructured electrode surface.

In this novel design, the CO_2_ gas-fed nozzle, through an adiabatic process, delivers CO_2_ in a triple point with all gas, liquid and solid phases. Solid CO_2_ (snow) has sufficient momentum to displace loose surface structures it collides with, while liquid CO_2_ is an excellent hydrocarbon solvent; finally, the CO_2_ gas is a non-reactive, heavier-than-air flow that provides additional surface cleaning via drag [24]. These three processes simultaneously contribute to a surface free from debris.

Two distinct CO₂-snow-assisted processing methods were evaluated in this study. The first approach, “tandem” CO₂ processing, involves directing the CO₂ stream simultaneously with the laser, aligning precisely with the laser’s interaction point on the Pt-10Ir electrode surface. This real-time cleaning process removes ablated particulates immediately as they form on the surface.

The second approach, “lag” CO₂ processing, involves restructuring the electrode area in smaller segments, with CO₂ cleaning applied after each segment is completed. Unlike the tandem method, the CO₂ stream does not interact with the laser beam, ensuring that restructuring occurs before CO₂ processing begins.

For comparison, ultrasonic cleaning was performed by fully submerging the HSR™ electrodes in ethanol and placing the beaker in an ultrasonic bath for one minute, allowing agitation to remove less-stable surface structures.

### Electrochemical Characterization

The electrochemical performance of the electrodes was evaluated using cyclic voltammetry (CV) and electrochemical impedance spectroscopy (EIS). These measurements were employed to quantify key performance metrics, including charge storage capacity (CSC), impedance, and specific capacitance (SC), providing a comprehensive assessment of each electrode’s electrochemical behavior and stability following different cleaning methods [1,14,17,26–28].

All electrochemical measurements were performed with a 5000E Interface Potentiostat (Gamry, Warminster, PA, USA) and a three-electrode test cell, consisting of an Ag/AgCl reference electrode, a coiled platinum wire counter electrode, and the working electrode being evaluated. Phosphate buffered saline (Blood Bank Saline, Azer Scientific, Morgantown, PA) was used as the conductive electrolyte medium.

CV tests were performed over a potential window of –0.6 V to 0.8 V with a voltage sweep rate of 50 mV/s. Charge storage capacity (CSC) was calculated as the time-integral of the current measured during cyclic voltammetry, normalized by the GSA of the working electrodes. Specific capacitance (SC) was extracted by fitting a Randles equivalent circuit model to the EIS data and normalizing the resulting capacitance to the electrodes’ GSA. All additional CV and EIS experimental procedures and setup details are consistent with those described in our previous studies [17,28].


$$\begin{array}{c} CSC=\frac{\;\text{1}}{GSA}\int Idt \end{array}$$ (1)

The electrochemical characterization process also serves as a means of surface agitation to evaluate the morphological stability of the electrodes. Repeated electrochemical cycling, particularly during cyclic voltammetry, imposes mechanical and electrochemical stresses on the electrode surface. This can induce irreversible morphological changes, especially in electrodes with unstable or weakly bonded surface features, thereby revealing the impact of different cleaning methods on long-term structural integrity [29,30].

The repeated cyclic voltammetry cycling, conducted for 45 cycles at a sweep rate of 50 mV/s, serves as the primary mechanism of surface agitation during electrochemical characterization. This testing protocol enables assessment of the electrodes’ ability to withstand electrochemical stresses with minimal morphological degradation. To evaluate stability, the surface morphology of electrodes is examined both before and after electrochemical cycling/agitation, allowing for direct comparison and identification of any changes induced by the different cleaning treatments. Electrochemical measurements were repeated on each of the three electrodes fabricated at each condition, with reported values being the average of the three repeats, and error bars representing their standard deviation.

### Morphological Characterization

The surface morphology of each electrode was analyzed before and after electrochemical characterization, both qualitatively and quantitatively, using a scanning electron microscope (SEM) (ZEISS, Oberkochen, Germany), which provided high-resolution imaging capable of capturing nanoscale surface features. Electrodes were mounted in the SEM without any metal coating. Because the bulk electrode material (Pt-10Ir) is highly conductive, no metal coating was necessary to achieve high fidelity SEM micrographs. No significant drift, distortion, or other charging artifacts were present during imaging.

3D, stereoscopic reconstruction of SEM images was utilized to enable a high-resolution evaluation of morphological changes, before and after surface agitation. SEM micrographs were captured at two tilt angles (0° and 15°) and processed using stereoscopic reconstruction software, MountainsSEM (Digital Surf, France) to generate 3D surface models. This method leverages the high lateral resolution of SEM for analyzing the fine, nanoscale changes in texture.

Surface morphology was quantitatively assessed using area roughness parameters in accordance with ISO 25178, specifically the root-mean-square height (S_q_) and the developed interfacial area ratio (S_dr_), as defined in Eq. (2) and Eq. (3), respectively. In these equations, *z(x,y)* represents the surface height at position relative to the mean reference plane and 𝐴 denotes the planar surface area of the analyzed region. Partial derivatives ∂𝑧/∂𝑥 and ∂𝑧/∂𝑦 describe local surface slopes used to calculate the true developed surface area. In this study, morphological stability is quantitatively defined as the change in S_q_ and S_dr_ values before and after surface agitation, with smaller variations in these parameters indicating greater structural stability of the electrode surface. Characterization was performed on each of the three electrodes fabricated at each condition, with reported values being the average of the three repeats, and error bars representing their standard deviation.

The highest fluence level (9.7 J/cm²) was excluded from this analysis, as the pronounced surface height variations introduced at that fluence significantly reduced the accuracy of stereoscopic 3D reconstruction which may lead to inconsistent or unreliable measurements.


$$\begin{array}{c} Sq=\;\sqrt{\frac{\text{1}}{A}\iint A\;Z^{\text{2}}(x,y)dxdy} \end{array}$$ (2)

$$\begin{array}{c} Sdr=\frac{\text{1}}{A}\left[\iint A\;\left(\sqrt{\left[\text{1}+{\left(\frac{\partial z(x,y)}{\partial x}\right)}^{\text{2}}+{\left(\frac{\partial z(x,y)}{\partial y}\right)}^{\text{2}}\right]}-\text{1}\right)dxdy\right] \end{array}$$ (3)

## Results and discussion

### Characterization of Electrode Morphology

**Fig 3:**
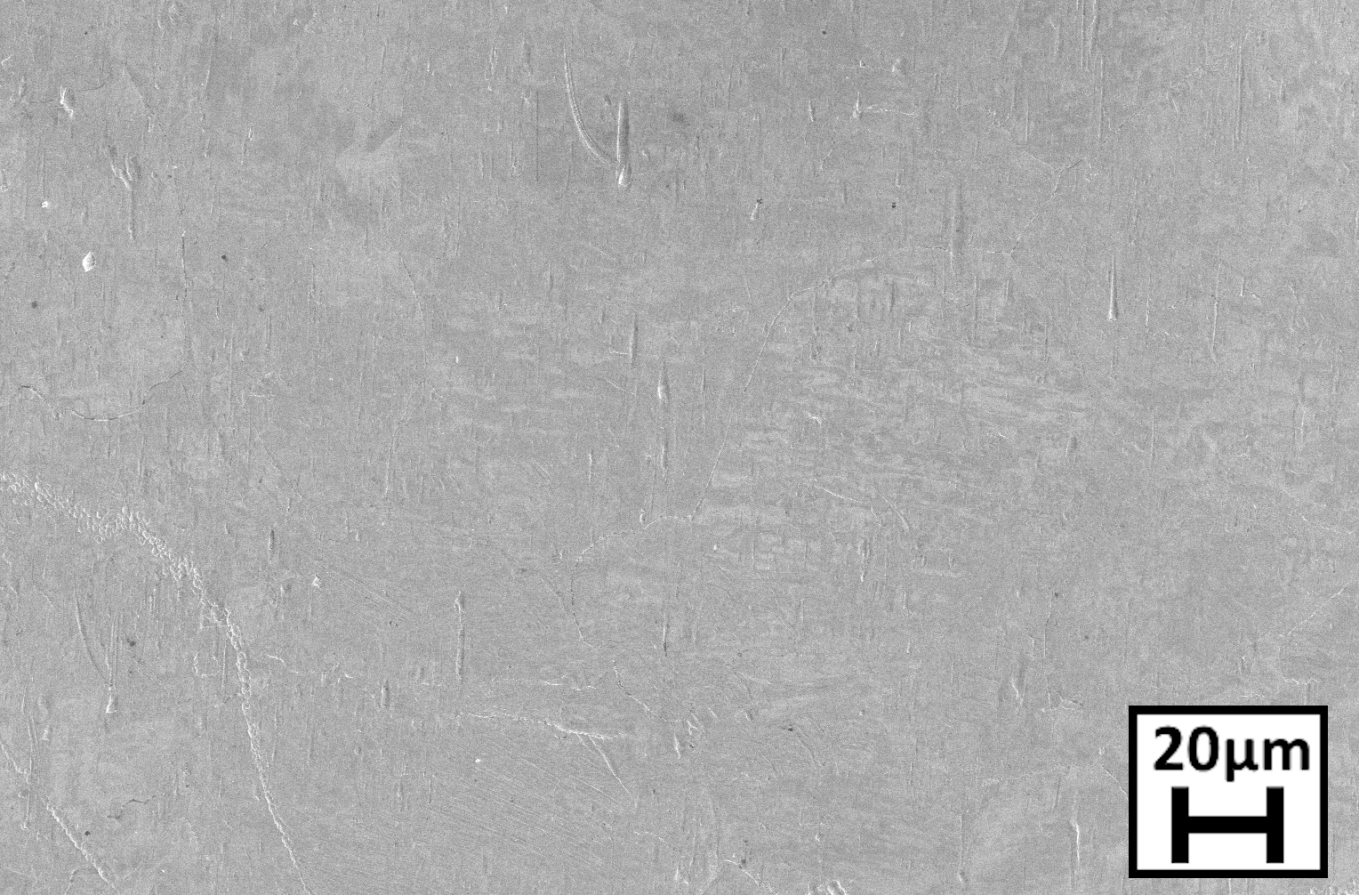
SEM micrograph of a pristine, unaltered Pt-10Ir electrode.

**Fig 4:**
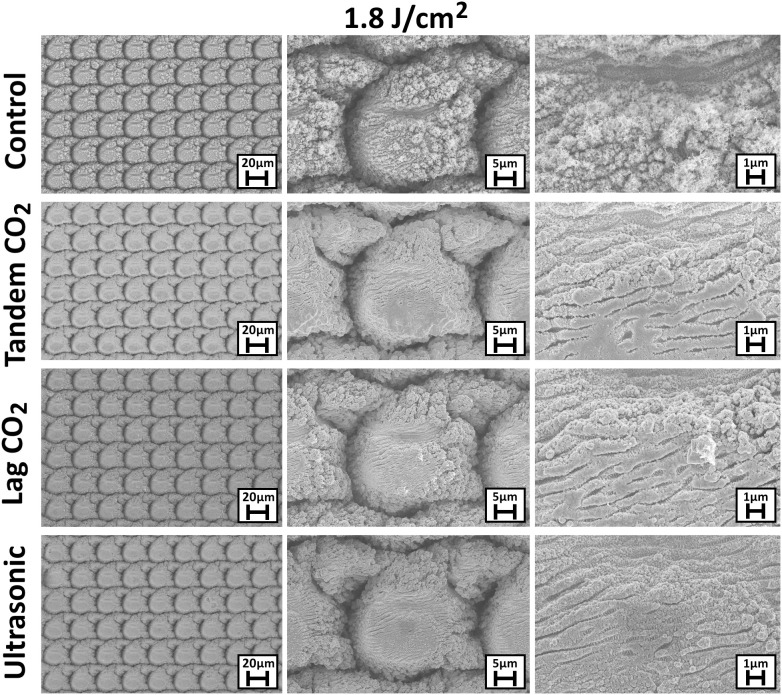
SEM micrographs of HSR™ electrodes fabricated at a laser fluence of 1.8 J/cm², comparing surface morphology across four processing conditions: Uncleaned control (1^st^ row), Tandem CO₂ cleaning (2^nd^ row), Lag CO₂ cleaning (3^rd^ row), and Ultrasonic cleaning (4^th^ row); First column micrographs were captured at 1kx magnification, highlighting overall surface morphology, while middle and right column micrographs were taken at 5kx and 20kx magnification to reveal nanoscale structures and surface detail.

The surface morphology of a pristine, unmodified Pt-10Ir electrode is shown in **Fig 3**, providing a baseline comparison for the laser-induced features. Electrodes fabricated via the HSR™ technique exhibit a highly textured, periodic peak-valley architecture across the restructured regions. At low fluence (1.8 J/cm²), the surface is characterized by coarse, pillar-like structures that are approximately circular in shape, with diameters ranging from 25 to 27 µm, and an interpillar spacing of 4–5 µm. These morphological features are clearly observable in the low-magnification SEM micrographs shown in **Fig 4**, highlighting the uniformity and periodicity of the laser-induced surface restructuring at this fluence level. The centers of the pillar structures appear less restructured than their perimeter, resulting in a relatively flat and uniform plateau across adjacent peaks. This plateau is roughly level with the surface as it was before restructuring. In contrast, the valleys between the pillars exhibit a higher degree of surface restructuring. Additionally, a secondary structure, approximately 10 µm in length is consistently observed near the top-right edge of each peak, suggesting directional effects in laser material ablation or redeposition.

**Fig 5:**
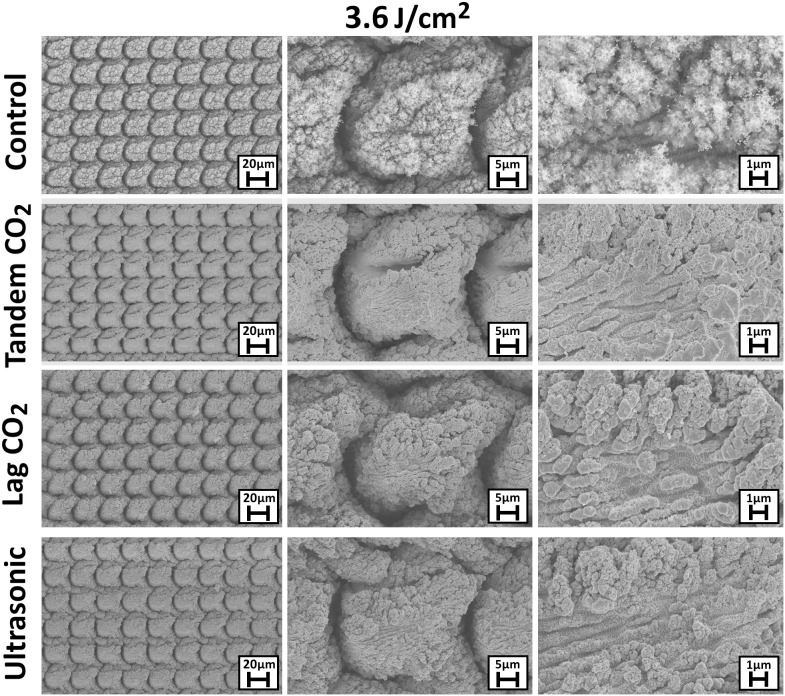
SEM micrographs of HSR™ electrodes fabricated at a laser fluence of 3.6 J/cm², comparing surface morphology across four processing conditions: Uncleaned control (1^st^ row), Tandem CO₂ cleaning (2^nd^ row), Lag CO₂ cleaning (3^rd^ row), and Ultrasonic cleaning (4^th^ row); First column micrographs were captured at 1kx magnification, highlighting overall surface morphology, while middle and right column micrographs were taken at 5kx and 20kx magnification to reveal nanoscale structures and surface detail.

**Fig 6:**
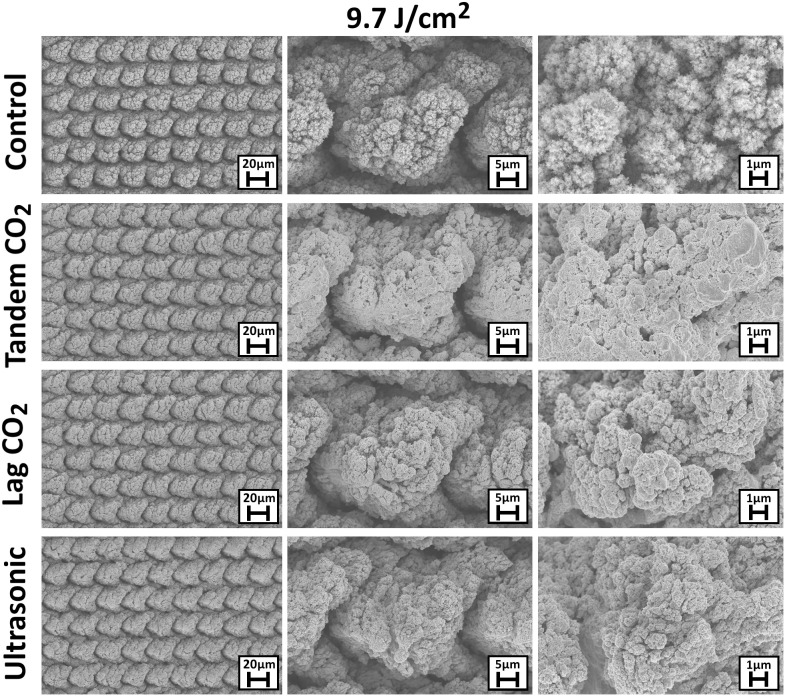
SEM micrographs of HSR™ electrodes fabricated at a laser fluence of 9.7 J/cm², comparing surface morphology across four processing conditions: Uncleaned control (1^st^ row), Tandem CO₂ cleaning (2^nd^ row), Lag CO₂ cleaning (3^rd^ row), and Ultrasonic cleaning (4^th^ row); First column micrographs were captured at 1kx magnification, highlighting overall surface morphology, while middle and right column micrographs were taken at 5kx and 20kx magnification to reveal nanoscale structures and surface detail.

As laser fluence increases to 3.6 J/cm² and 9.7 J/cm² (**Fig 5** and **Fig 6**), the rate of material ablation intensifies, resulting in wider valleys between adjacent pillar structures. While the interpillar spacing in the north and south (relative to the orientation of the micrographs) directions remains relatively constant at 4–5 µm, the spacing in the east and west direction expands to approximately 8–10 µm, indicating deeper surface restructuring. At these higher fluences, the pillars lose their distinct circular shape, and the previously observed secondary structures become indistinguishable, merging into a single, more continuous surface topography.

As laser fluence increases, the electrode surfaces become more aggressively restructured, exhibiting greater roughness and fine-scale texture across most of the pillar structures. At 3.6 J/cm², remnants of lightly restructured regions can still be observed near the centers of the peaks, similar to those seen at the lowest fluence. However, at 9.7 J/cm², these central unmodified areas are entirely absent, indicating a fully restructured and uniformly textured surface as a result of the higher ablation energy.

At higher magnifications, the nanoscale structures that densely populate the surface of the control electrodes become clearly visible. As laser fluence increases, the density and distribution of these fine features also increase, progressively covering a larger portion of the restructured area. This suggests that higher fluence not only intensifies the coarse-scale restructuring but also promotes the formation and/or redeposition of finer particulate structures across the surface.

All cleaning methods examined in this study result in distinct surface morphologies compared to the uncleaned control electrodes. The most prominent difference is the removal of loose nanoparticles, exposing the underlying, more stable microstructures. At 1.8 J/cm², the cleaned electrodes exhibit a consistent wave-like pattern across the surface of the peak structures, indicating that while the finer particulates are effectively removed, the integrity of the primary hierarchical features is preserved across all cleaning approaches.

Variations between cleaning methods is more pronounced at higher fluence levels, where the effects of cleaning are magnified by the increased randomness and complexity of the surface textures. Among the cleaning methods, Tandem CO₂ processing produced the most distinct surface morphology, characterized by a generally smoother texture, particularly towards the south of the pillar structures. In Tandem CO₂ processing, the CO₂ stream is applied simultaneously with the femtosecond laser at the focal point during restructuring.

This simultaneous application of the CO₂ stream during femtosecond laser restructuring alters the fundamental nature of the laser–material interaction and can influence the dynamics of ablation, restructuring, and redeposition through multiple coupled mechanisms.

First, the introduction of the CO₂ stream drastically lowers the local temperature of the targeted regions of the electrode surface, which may impede ablation and/or restructuring by limiting the thermal energy available for efficient material removal. In parallel, dry-ice particles and transient liquid CO₂ condensation within the interaction zone may absorb, scatter, or reflect a fraction of the incident laser energy, producing a shielding effect that attenuates the effective fluence delivered to the surface [25]. By measuring the laser fluence after the point of interaction with the CO_2_ stream, we can observe an average 16% reduction in effective laser fluence, across the range of fluences used in this study. Collectively, these thermal and optical attenuation mechanisms reduce the net energy contributing to ablation and restructuring, resulting in less aggressive material removal and overall smoother surface morphologies.

This CO₂ shielding effect filters out lower-energy portions of the beam, thereby modifying the energy profile of the laser–Pt-10Ir interaction. As a result, the surfaces produced with Tandem CO₂ cleaning exhibit characteristics typically associated with lower-fluence restructuring.

This effect is most apparent in the Tandem CO₂-treated electrode shown in **Fig 5**, where a crevice-like feature at the northern portion of the pillar is present at 3.6 J/cm^2^, while it is absent for other processing methods at the same fluence. This feature is ubiquitous across all methods at the lower fluence in **Fig 4**, but disappears as fluence increases. Its persistence under tandem CO_2_ processing indicates a reduction of the effective fluence, and delayed material ablation in that region of the pillar.

Additionally, this crevice is present with all treatments at 1.8 J/cm^2^ (**Fig 4**), but appears shallower under tandem CO_2_ processing, indicating attenuated effective fluence. The reduced depth of this feature is evident through the milder contrast in brightness between the crevice floor and surrounding material and is most apparent at the 20kx magnification.

Furthermore, the low temperature of the CO₂ stream may transiently embrittle fine surface features, particularly loose micro- and nanostructures generated during laser restructuring. While the laser locally delivers high thermal energy to the electrode surface, rapid cooling induced by the CO₂ stream may reduce the mechanical toughness of these features. Under such conditions, the combined action of CO₂-induced embrittlement and aerodynamic drag may facilitate the preferential removal of fragile or weakly adhered structures that would otherwise persist or redeposit onto the surface. This mechanism provides a plausible explanation for the observed reduction in fine-scale surface texture under Tandem CO₂ processing.

Directional effects are also evident in the resulting morphologies. In particular, the south-facing side of the pillar structures exhibit noticeably smoother surfaces compared to the opposite side. We hypothesize that this asymmetry arises from the drag force of the CO₂ stream acting most strongly on regions directly exposed to the flow while the material is in a transiently excited, mechanically weakened, or locally embrittled state during laser interaction. This real-time interaction between the CO₂ stream and the evolving surface may further suppress the formation or redeposition of fine features. Because the influence of the CO₂ stream is strongly dependent on local geometry, this effect is most pronounced near the pillar peaks on the south face, where exposure to the CO₂ flow and associated drag forces is greatest.

In contrast, both Lag CO₂ processing and ultrasonic cleaning are post-fabrication processes that act solely on the surface after laser structuring is complete, and therefore, do not influence the laser-induced material response during ablation.

**Fig 7:**
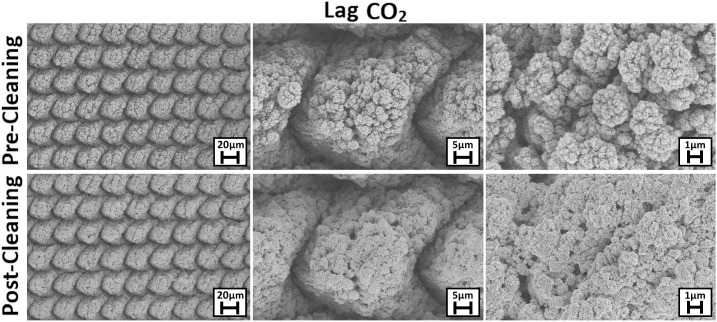
SEM micrographs of HSR™ electrodes fabricated with Lag CO_2_ cleaning at a laser fluence of 9.7 J/cm², demonstrating the effect of the cleaning process on the surface; First column micrographs were captured at 1kx magnification, highlighting overall surface morphology, while middle and right column micrographs were taken at 5kx and 20kx magnification to reveal nanoscale structures and surface detail.

**Fig 8:**
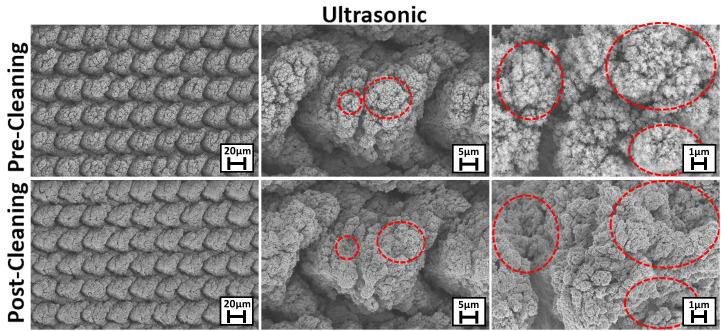
SEM micrographs of HSR™ electrodes fabricated with ultrasonic cleaning at a laser fluence of 9.7 J/cm², demonstrating the effect of the cleaning process on the surface; First column micrographs were captured at 1kx magnification, highlighting overall surface morphology, while middle and right column micrographs were taken at 5kx and 20kx magnification to reveal nanoscale structures and surface detail. Red dashed circles highlight some prominent surface structures that were removed as a result of ultrasonic cleaning.

Although the electrodes produced with Lag CO_2_ and ultrasonic cleaning may appear similar, their different effects on the surface are most apparent when examining the morphology of the same location, taken before and after cleaning, as shown in **Fig 7** and **Fig 8**. Electrodes produced at 9.7 J/cm² were examined, because differences were most pronounced. As previously discussed, both methods effectively remove nanoparticles from the surface of the electrode, but this direct comparison reveals how they alter the larger and more stable microstructure.

**Fig 7** shows the effect of Lag CO_2_ on the electrode surface. When examining the 5kx and 20kx micrographs before and after cleaning, it is clear that the process removes some of the larger, underlying microstructures, as large as 2–4 µm in length. **Fig 8** shows that ultrasonic cleaning also removes these underlying microstructures, but the structures removed are as large as 5–10 µm. This indicates that ultrasonic cleaning is more destructive to the larger and more stable microstructure, creating significant changes in the surface morphology.

All cleaning methods alter the underlying electrode surface to varying extents, but the key distinction between them is if they primarily remove already unstable surface structures, or also excessively damage the underlying hierarchical structures. By analyzing the impact of cleaning on electrode morphology, we can observe that Lag CO_2_ preserves more of the stable meso-structures, while ultrasonic cleaning is more damaging. As we investigate later in this report, this excessive destruction of underlying hierarchical structures correlates to reduced electrochemical performance.

### Characterization of Electrode Morphological Stability

**Fig 9:**
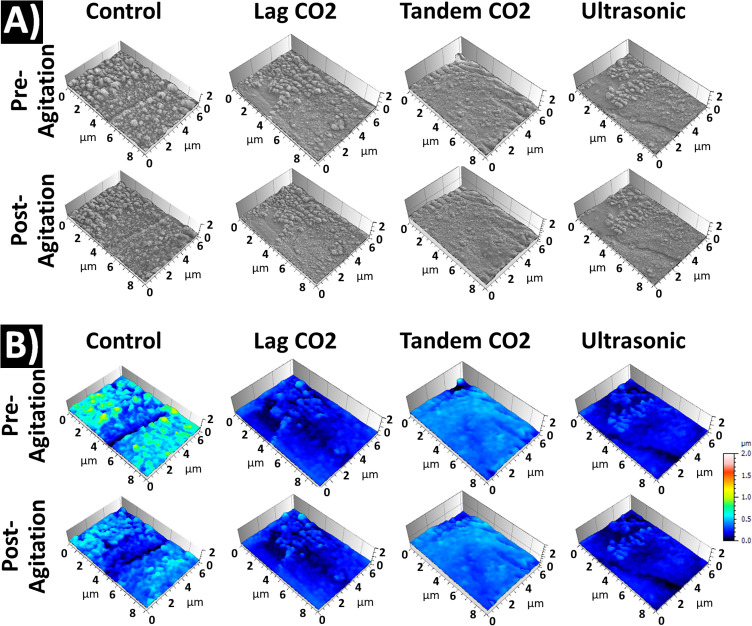
A) 3D SEM stereoscopic reconstructions of representative areas of HSR™ electrode surfaces processed with each cleaning method, before and after electrochemical agitation. **B)** Height maps of stereoscopic reconstructions of representative areas of HSR™ electrode surfaces processed with each cleaning method, before and after electrochemical agitation.

**Fig 10:**
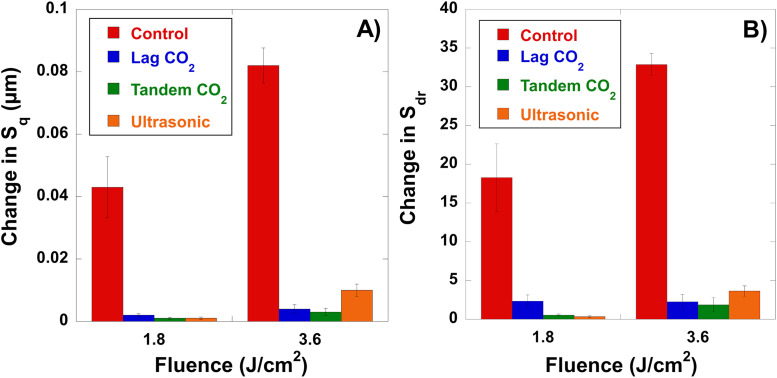
A) The change in root mean square height (S_q_) of electrode surfaces produced at 1.8 and 3.6 J/cm^2^, before and after agitation. **B)** The change in developed interfacial area ratio (S_dr_) of electrode surfaces produced at 1.8 and 3.6 J/cm^2^, pre- and post-agitation. Each datapoint represents the average value of three electrodes produced at each condition. Error bars represent the standard deviation.

As shown in the SEM micrographs presented in the previous section, all processing and cleaning methods evaluated were effective in removing less stable nanoparticles from the electrode surface. However, the key question remains: *how does the removal of these surface structures* *influence the electrodes’ morphological stability*? To address this, 3D stereoscopic reconstructions were generated from SEM micrographs captured before and after electrochemical characterization (agitation) and are shown in **Fig 9**. With the availability of 3D height data, the mechanical stability of the electrodes can be quantitatively assessed by analyzing changes in surface roughness parameters, specifically root-mean-square height (S_q_) and developed interfacial area ratio (S_dr_). These parameters offer insight into the extent of surface alteration resulting from electrochemical agitation. As illustrated in **Fig 10**, both S_q_ and S_dr_ change similarly after agitation, and therefore will be discussed collectively to evaluate the relative stability imparted by each cleaning method.

**Fig 11:**
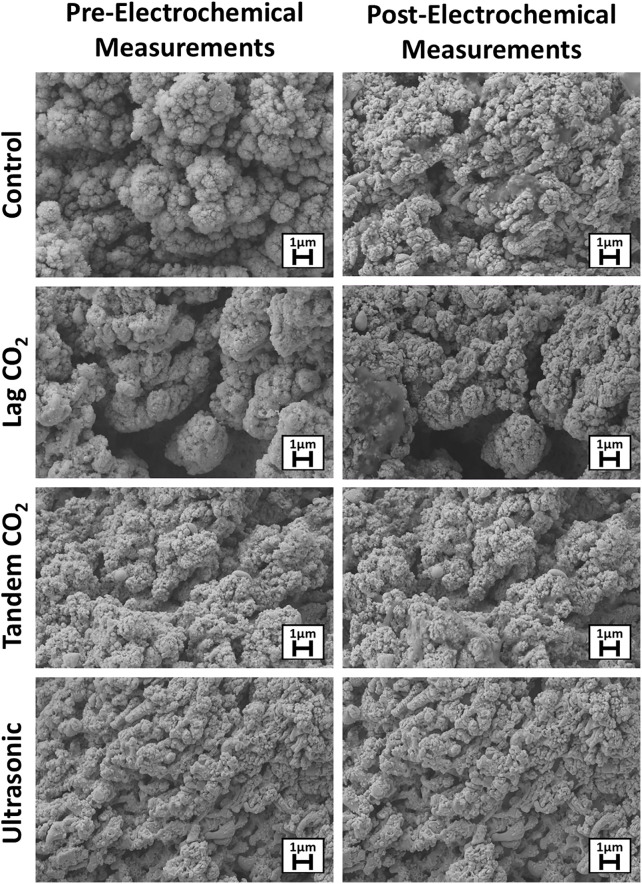
SEM micrographs of HSR™ electrodes fabricated at a laser fluence of 9.7 J/cm², comparing surface morphology across four processing conditions, before and after being agitated by electrochemical measurements. All micrographs are captured at 20kx magnification.

As previously discussed, the accuracy of this surface quantification technique diminishes for surfaces with severe variations in height, and was not performed on electrodes produced at 9.7 J/cm^2^ fluence. In place of quantitative analysis, **Fig 11** presents SEM micrographs taken of electrodes produced at this fluence, before and after electrochemical testing, to provide a qualitative comparison of surface stability across the different cleaning methods.

The control electrodes exhibited substantial changes in both S_q_ and S_dr_ values following electrochemical cycling, clearly indicating a high degree of morphological instability. These shifts are primarily due to the detachment of nanoparticles and other weakly adhered surface structures, which are easily dislodged during electrochemical agitation. In contrast, electrodes treated with any of the three cleaning methods demonstrated markedly smaller changes in these surface parameters, confirming their enhanced morphological stability and improved resistance to surface degradation under repeated cycling conditions.

For electrodes fabricated at 1.8 J/cm², the Lag CO₂ processing method exhibited the greatest change in surface parameters among the cleaned electrodes; however, all cleaning methods at this fluence resulted in relatively stable surfaces, with only minimal changes in roughness. As the fluence increased to 3.6 J/cm², a general rise in morphological instability was observed across all electrodes. At this higher fluence, electrodes cleaned using ultrasonic methods showed slightly greater instability compared to those treated with Tandem or Lag CO₂ processing, suggesting that CO₂-based approaches are more effective in preserving surface integrity under more aggressive restructuring conditions.

This slightly increased instability in ultrasonically cleaned electrodes at higher fluence may be attributed to the loosening of less-permanent microstructures during the cleaning process. These features, which are more prevalent at higher fluence levels, may become more susceptible to detachment during subsequent electrochemical agitation. Despite this, all cleaning methods demonstrated a notable improvement in morphological stability compared to the control electrodes, reinforcing the conclusions drawn from the qualitative analysis and highlighting the effectiveness of preemptive surface cleaning/processing in stabilizing HSR™-fabricated electrodes.

These trends are largely corroborated by SEM micrographs in **Fig 11**. Although **Fig 11** only shows electrodes produced at 9.7J/cm^2^, the effect of electrochemical agitation on each type of electrode is representative of those at lower fluences. The morphology of the control electrode changes dramatically after electrochemical measurements, with most of the fine nanostructures being removed from the surface. This corresponds to the major shift in S_q_ and S_dr_ shown in **Fig 10**. When a cleaning method is used, there is an evident improvement in morphological stability. Electrode surfaces before and after agitation are practically identical, reflecting the corresponding reduced instability of S_q_ and S_dr_. However, there is no apparent difference in the stability of ultrasonically cleaned electrodes compared to CO_2_ cleaned electrodes. All post-measurement electrodes sporadically have possibly amorphous, gray features across their surfaces. These features are not intrinsic part of electrode morphology but are residue from the saline used during electrochemical measurements.

### Electrochemical Performance Characterization

**Fig 12:**
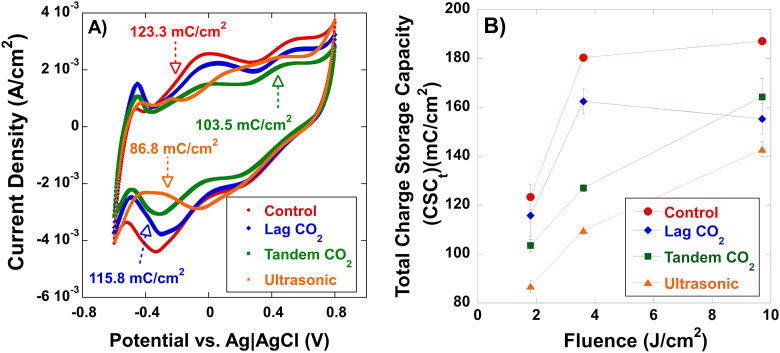
A) Cyclic voltammograms of various processing methods and control electrodes restructured at 1.8 J/cm^2^, taken from—0.6V to 0.8V, against an Ag/AgCl electrode. Labels indicate corresponding total charge storage capacity (CSC_t_) for each voltammogram. **B)** Total charge storage capacity (CSC_t_) for electrodes restructured at 1.8 J/cm^2^, 3.6 J/cm^2^, and 9.7 J/cm^2^ across each cleaning/processing method and a control electrode. Each datapoint represents the average value of three electrodes produced at each condition. Error bars represent the standard deviation.

To assess the electrochemical performance of the electrodes, cyclic voltammograms were analyzed and compared against those of an uncleaned control electrode, as shown in **Fig 12a**. This comparison provides insight into how each cleaning method influences key performance metrics, such as charge storage capacity. The total charge storage capacity (CSCₜ) was calculated by integrating the area enclosed by the cyclic voltammograms with respect to time and normalizing the result by the exposed geometric surface area (GSA) of the electrodes. This metric provides a quantitative measure of the electrodes’ ability to store charge, and the results are presented in **Fig 12b**.

Across all cleaning methods, electrodes exhibit a generally logarithmic, positive correlation between CSCₜ and laser fluence, reflecting the enhanced surface texture and increased electrochemical surface area (ESA) achieved at higher fluence levels. As fluence increases, so does electrochemical performance, particularly due to the more aggressive restructuring and greater surface texture. However, at the highest fluence tested (9.7 J/cm²), the performance gains begin to plateau or diminish—most notably in the case of Lag CO₂ processing, where CSCₜ shows a slight decline. This suggests that beyond a certain threshold, additional surface roughening may introduce structural inefficiencies or instability that limits further performance improvements.

In general, uncleaned electrodes exhibit the highest CSCₜ values, likely due to the presence of loosely bound nanoparticles that contribute additional surface area. This is followed by electrodes cleaned with Lag CO₂, Tandem CO₂, and finally ultrasonic cleaning, which consistently shows the lowest CSCₜ across most fluence levels. However, at the highest fluence (9.7 J/cm²), this trend shifts slightly: Tandem CO₂ outperforms Lag CO₂, likely due to its ability to preserve more of the functional surface morphology while effectively removing unstable structures.

While all cleaned electrodes exhibit a lower initial CSCₜ compared to the uncleaned control, this difference must be interpreted in the context of long-term performance stability. The higher CSCₜ of control electrodes is largely due to the presence of fine nanoparticles, which elevate the electrochemical surface area (ESA). However, as previously discussed, these unstable features are prone to detachment during operation, likely leading to a decline in performance. In contrast, cleaned electrodes are expected to maintain more consistent performance over time, as their surfaces are composed of more durable and structurally stable structures.

**Fig 13:**
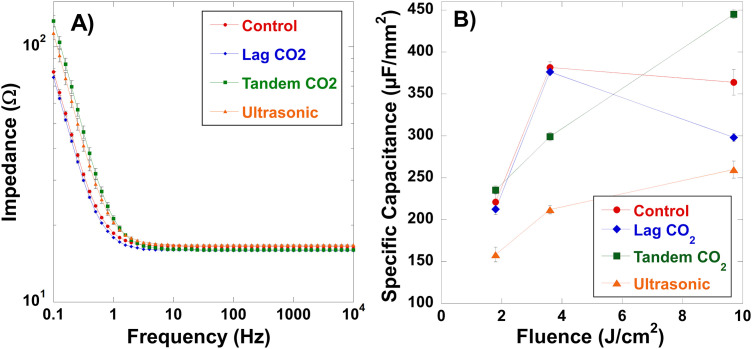
A) Bode plots of impedance magnitude as a function of frequency (plotted in the 0.1-10^4^ Hz frequency range) for electrodes restructured at 1.8 J/cm^2^ across each cleaning method and a control. **B)** Specific capacitance for electrodes restructured at 1.8 J/cm^2^, 3.6 J/cm^2^, and 9.7 J/cm^2^ across each cleaning method and a control. Each datapoint represents the average value of three electrodes produced at each condition. Error bars represent the standard deviation.

Electrochemical impedance spectroscopy (EIS) was used to measure electrodes’ impedance across a frequency range of 0.1 to 10⁴ Hz, with results displayed as Bode plots in **Fig 13a**. At 1 kHz—a standard reference frequency for neural stimulation—the Lag CO₂ and Tandem CO₂ processed electrodes demonstrated comparable impedance values. The control and ultrasonically cleaned electrodes also showed similar impedance values, though slightly higher than those of the CO₂-treated electrodes. While the differences are subtle and difficult to distinguish visually in the plot due to the overall similarity in impedance profiles, the relative impedance ranking at 1 kHz from highest to lowest is: Lag CO₂, Tandem CO₂, Control, and Ultrasonic.

The EIS data were fitted to a Randles equivalent circuit model to extract the specific capacitance of the electrodes, as shown in **Fig 13b**. The results reveal a generally logarithmic increase in specific capacitance with rising laser fluence, consistent with trends observed in CSCₜ measurements. Across all fluence levels tested, ultrasonically cleaned electrodes consistently exhibited the lowest specific capacitance, aligning with their lower CSCₜ values and reflecting the possible loss of beneficial surface nanostructures during the CO_2_-snow assisted process.

The control and Lag CO₂-processed electrodes generally exhibit comparable specific capacitance values, both consistently outperforming the ultrasonically cleaned electrodes. The performance of Tandem CO₂-processed electrodes, however, shows greater variability across fluence levels. Notably, they outperform all other electrodes at 1.8 and 9.7 J/cm², but underperform at 3.6 J/cm². Interestingly, the capacitance trend for Tandem CO₂ electrodes increases in a more linear fashion with fluence, contrasting with the logarithmic trends observed in other groups. This linear relationship mirrors the behavior previously noted in the CSCₜ results (**Fig 12b**), suggesting that the Tandem CO₂ process may modify the laser-material interaction in a more uniform and scalable manner. We propose this behavior can be explained by two coupled phenomena.

In ultrafast laser machining of metals, the rate of ablation increases logarithmically as a function of fluence, but saturates past a certain, material-dependent threshold [31]. Above this threshold, a greater fraction of laser energy is dissipated into the material, which manifests greater heat affected zone, but does not ablate more material. In terms of energy efficiency, this maximum, saturated rate of ablation for most metals occurs between 0.5–2 J/cm^2^.

This intrinsic saturation is aggravated by a second, plasma shielding effect. At higher fluences, a plume of laser-generated plasma is ejected above the target area, absorbing or reflecting incoming laser energy and reducing the effective fluence. Simultaneously this ejected material can redeposit into the newly ablated pores and gaps, partially refilling laser-formed features and limiting accessible surface area as fluence increases.

Together, these two effects lead to diminishing returns in ablation rate, and consequently in surface area and performance, as fluence increases. This trend is reflected by the performance data in **Fig 12b** and **Fig 13b**, although the threshold appears to occur at a higher fluence (~3.6 J/cm^2^), likely due to Pt-10Ir being significantly harder than the metals studied in the literature [31].

We hypothesize that tandem CO_2_ delays the onset of this saturation threshold. First, the intrinsic ablation-efficiency limit remains, but is shifted to a higher nominal fluence, due to CO_2_ induced shielding which attenuates the effective fluence. Second, the fluence-dependent plasma shielding and redeposition are largely mitigated, because the CO_2_ stream continuously removes the plume of ablated material.

Tandem CO_2_ leads to smoother surfaces and thereby initially reduces performance, in part due to its shielding effect. However, unlike plasma shielding, CO_2_-induced shielding does not scale with laser fluence. Consequently, at a sufficiently high fluence, the suppression of plasma shielding outweighs the CO_2_ shielding, allowing for a net increase in the maximum ablation rate, surface area and electrochemical performance, compared to the control.

If this trend continues beyond the tested fluence range, Tandem CO₂-snow assisted processing could raise the upper performance threshold of HSR™ electrodes—though further experimental validation at higher fluences would be necessary to confirm this hypothesis.

Interestingly, ultrasonically cleaned electrodes show a similar, linear trend in performance. However, the ultrasonic cleaning leads to a unilateral decrease in performance that is more significant than tandem CO_2_ processing. Furthermore, it is a post-process which functions based on an entirely different mechanism which does not affect the laser-material interaction and does not overcome the saturation of the ablation rate. Therefore, there is insufficient evidence to suggest that extrapolating this trend to higher fluences would result in a net performance increase for ultrasonically cleaned electrodes.

## Concluding remarks

This study demonstrates that preemptive surface cleaning and surface processing of femtosecond laser hierarchically restructured electrodes—through the removal of loose and impermanent nanostructures—significantly enhances their morphological stability under mechanical and electrochemical stresses. Both CO₂-snow-assisted processing and ultrasonic cleaning improved stability relative to untreated control electrodes; however, CO₂-snow-assisted technique offered notable advantages in preserving electrochemical performance. Unlike ultrasonic and other post-processing methods, CO₂-snow-assisted processing can be implemented in-operando and in tandem with the HSR™ fabrication process, enabling real-time particulate removal with no additional manufacturing time. This integration supports efficient, scalable production of structurally stable, high-performance electrodes for neural interfacing applications. Moreover, CO₂-snow-assisted processing is significantly less abrasive than ultrasonic cleaning, more effectively maintaining the integrity of both meso-scale and micro-scale surface structures. As a result, CO₂-snow processed electrodes exhibited smaller reductions in charge storage capacity, and specific capacitance, and a smaller increase in impedance compared to controls, particularly at higher fluence levels. Tandem CO₂ processing uniquely demonstrated a more linear relationship between fluence and performance, and outperformed all other groups in specific capacitance at the highest fluence tested (9.7 J/cm²). These results suggest Tandem CO_2_’s potential to enable continued scalable gains in electrode performance beyond their performance limit without any additional processing time. While this study confirms the mechanical and electrochemical advantages of CO₂-snow-assisted processing, future studies are needed to determine whether these benefits translate into long-term device stability in realistic operating conditions. Additional investigations should explore extended cycling durability, and in-vivo performance to validate these findings for clinical or commercial neural interfacing applications.
